# Food and Drug Administration Database Secondary Analysis: Difference in Operative Hysteroscopy Device Adverse Event Reporting

**DOI:** 10.1016/j.jmig.2025.04.009

**Published:** 2025-05-06

**Authors:** Arielle N. Valdez-Sinon, Anja S. Frost, Anita M. Madison, Rand El Sharaiha, Kristin E. Patzkowsky, Megan E. Gornet

**Affiliations:** Department of Gynecology and Obstetrics, Johns Hopkins University School of Medicine, Baltimore, Maryland; Division of Minimally Invasive Gynecologic Surgery, Johns Hopkins University School of Medicine, Baltimore, Maryland; Division of Reproductive Endocrinology and Infertility, Johns Hopkins University School of Medicine, Baltimore, Maryland; Fertility and Reproductive Medicine Center, Washington University School of Medicine, St Louis, Missouri; Division of Minimally Invasive Gynecologic Surgery, Johns Hopkins University School of Medicine, Baltimore, Maryland; Fertility and Reproductive Medicine Center, Washington University School of Medicine, St Louis, Missouri

**Keywords:** MAUDE, TruClear, MyoSure, Resectoscope, Hysteroscopic morcellator

## Abstract

**Study Objective:**

To investigate, describe, and compare adverse event reports (AERs) and their patterns amongst commonly used operative hysteroscopy devices.

**Design:**

A secondary analysis of the Manufacturer and User Facility Device Experience (MAUDE) published by the Food and Drug Administration.

**Setting:**

N/A.

**Patients or Participants:**

Women who underwent hysteroscopic surgery, with adverse events reported to MAUDE.

**Interventions:**

Search terms within the MAUDE database included “resectoscope,” “hysteroscopic reciprocating morcellator,” “MyoSure,” and “TruClear.” Reports were categorized by device type, patient complications, and required interventions. Statistical analysis utilized Fisher’s exact tests.

**Measurements and Main Results:**

Between January 2014 and April 2024, 1872 AERs were identified for hysteroscopes: 664 for resectoscopes and 1208 for morcellation devices (MyoSure, *N* = 645 and TruClear, *N* = 563). While absolute complication rates are not able to calculated from MAUDE, there were significant differences in the reporting of patient complications: resectoscope devices had higher frequency of infection (p < .01) while morcellation devices had higher frequency reporting of hemorrhage (p < .001), uterine perforation (p < .001), and bowel perforation (p < .001). Morcellation device AERs more often reported operative intervention (1.1% vs 12.4%, p < .001). Subgroup analysis comparing AERs of morcellation devices showed the majority (73.2%) of TruClear AERs registered no patient impact or harm, while only 21.2% of MyoSure AERs reported no patient impact. MyoSure device AERs had higher frequency of hemorrhage (p < .001), infections (p < .001), uterine perforations (p < .001), and bowel perforations (p < .001). Additionally, MyoSure AERs reported more surgical intervention compared to TruClear AEs (19.5% vs 4.3%, p < .001).

**Conclusion:**

While conclusions within the MAUDE database are limited, especially given the lack of data concerning the volume of surgeries done with each device and the voluntary reporting mechanism, there are significant differences in the types of adverse events reported among operative hysteroscopy instruments. Morcellation AERs had a significantly higher frequency of patient complications and described more surgical interventions compared to AERs for resectoscopes. When comparing MyoSure and TruClear, MyoSure AERs described a significantly greater proportion of serious patient complications compared to TruClear device AERs. Devices with similar functions may differ in how stakeholders report complications.

## Introduction

Operative hysteroscopy is a common gynecologic procedure used for the diagnosis and treatment of a multitude of clinical presentations, including abnormal uterine bleeding, infertility, and postmenopausal bleeding. Intrauterine pathology, such as polyps and fibroids, can be removed with multiple different types of surgical equipment, such as mechanical morcellators (also known as tissue removal systems), resectoscopes, polyp forceps, or bipolar snares. While the selection of which equipment to use for removal of intrauterine pathology is ultimately per surgeon preference and/or device availability, previous studies have specifically compared the efficacy of resectoscopes to morcellators [1–4]. While three of these studies found no significant difference between these two devices in the removal of intrauterine pathology [1,3,4], one meta-analysis reported superior removal of intrauterine pathology with morcellators compared to resectoscopes [2]. A statistically significant reduction in operative time (<5 minutes) with hysteroscopic morcellators compared to resectoscopes has been reported [1–5].

Studies examining differences in the burden of complications and side effects associated with the use of resectoscopes compared to morcellators are similarly mixed. While there are considerations that may confer unique risks with each device (e.g., distention media choice based upon device energy type), a 2018 meta-analysis of five studies comparing resectoscopes and morcellators did not find a difference in complication rate [2]. To our knowledge, a more contemporary analysis comparing the safety risks of different operative hysteroscopy devices and their adverse events has not been published. National surveillance and reporting of adverse events associated with medical devices is published by the Food and Drug Administration (FDA) and can be publicly accessed via the Manufacturer and User Facility Device Experience (MAUDE). MAUDE is a voluntary reporting system and database for medical device reports whereby reports can be made by manufacturers, importers and device user facilities, health care professionals, patients, and consumers. Though many aspects of this database (i.e., inconsistent reporting, lack of formalized policing, inability to obtain the absolute volume of cases performed which is necessary to calculate absolute rates) limit the conclusions that can be made from comparing adverse events, the MAUDE database provides the unique opportunity to obtain data on rare-occurring complications.

With the significant increase in the number of hysteroscopic devices and procedures over time (with a projected growth rate of 7.5% for the hysteroscopy market from 2024 to 2030 [6]), an updated assessment of differences in safety profiles—or the occurrence of specific types of adverse events—between devices is warranted. Recent studies comparing operative hysteroscopy devices have not included operative complications as a primary outcome to guide clinical practice and personalized care. As significant surgical complications can occur during hysteroscopy, it is important for surgeons to be aware of any complication profile differences amongst hysteroscopy instruments for removing intrauterine pathology, as well as to understand the reporting systems—such as MAUDE—that exist to improve care.

In order to examine the reporting of adverse events among common devices used for an increasingly performed procedure, we performed a secondary analysis of adverse event reports (AERs) registered within MAUDE that were associated with the use of operative hysteroscopy devices. The objective of this study was to identify differences in reporting patterns of adverse events registered to MAUDE, as well as examine the differences in the proportions of these adverse events within AERs between resectoscopes and mechanical morcellators used for operative hysteroscopy.

## Methods

The MAUDE database is searchable for adverse events reported over the last 10 years. The database was queried for events occurring from 2014 to April 2024 using the following search terms: “resectoscope,” “hysteroscopic reciprocating morcellator,” “TruClear,” and “MyoSure.” Reports generated from MAUDE contain information for each event, including the date of the event, the specific device used, the device problem, and any patient impact. Every event report includes a descriptive narrative written by the reporter that provides further details on the event. Each event report was reviewed individually by one study team member. Duplicate and erroneously coded (i.e., none of the intended instruments were utilized) events were excluded. Events mentioning male patients or prostate- or urologic-related procedures were also excluded from analysis. The senior author was available for additional review of cases as needed. Each AER description was then secondarily categorized by the following: adverse event type, patient complication, and any secondary surgical intervention based on the reported primary complication. Of note, an AER registered within MAUDE does not always involve a patient complication or impact. For example, an AER may be recorded for a device that is noted to be malfunctioning prior to any patient use.

The submission of a majority of resectoscope AERs lacked detail on the energy type (monopolar or bipolar) for that device. Given the inability to appropriately categorize subtypes of resectoscopes, monopolar and bipolar resectoscopes were bundled for the study. While it was difficult to distinguish the resectoscope type, AERs for morcellators more clearly distinguished device type, allowing for the investigation of specific morcellator brands (TruClear and MyoSure).

Of note, the MAUDE database does not include the total number of devices sold or total number of cases performed. Therefore, our statistical analysis cannot provide information pertaining to the absolute rate of adverse events per unit case or per unit instrument sold. Instead, we used descriptive statistics as well as univariate comparative methods to describe adverse event rates where the numerator is represented by the adverse event in question and the denominator represents the total adverse events reported (total AERs). In this way, we are calculating proportions of adverse events within the MAUDE database but not proportions or frequencies of adverse events within the realm of total cases performed in clinical practice.

All categorical data were compared for statistical significance using Fisher’s Exact Tests based on appropriate sample size.

## Results

From January 2014 to April 2024, the preliminary MAUDE query yielded the following AERs: 1458 for resectoscopes, 813 for MyoSure, 602 for TruClear. After review and exclusion of irrelevant reports, a total of 1872 AERs were identified related to operative hysteroscopy devices: 664 AERs for resectoscopes and 1208 AERs for hysteroscopic morcellators (563 with TruClear devices and 645 with MyoSure devices). The total number of AERs registered each year amongst the devices is reflected in Table S1. While the largest number of TruClear AERs occurred in 2017 (189/563), 2023 was the year with the most resectoscope AERs (322/664). MyoSure AERs were more evenly distributed, with 2021 having the most AERs (81/645). For the initial analyses performed, the AERs for TruClear and MyoSure devices were pooled into a combined “morcellator” group (1208 events total).

**Fig. 1:**
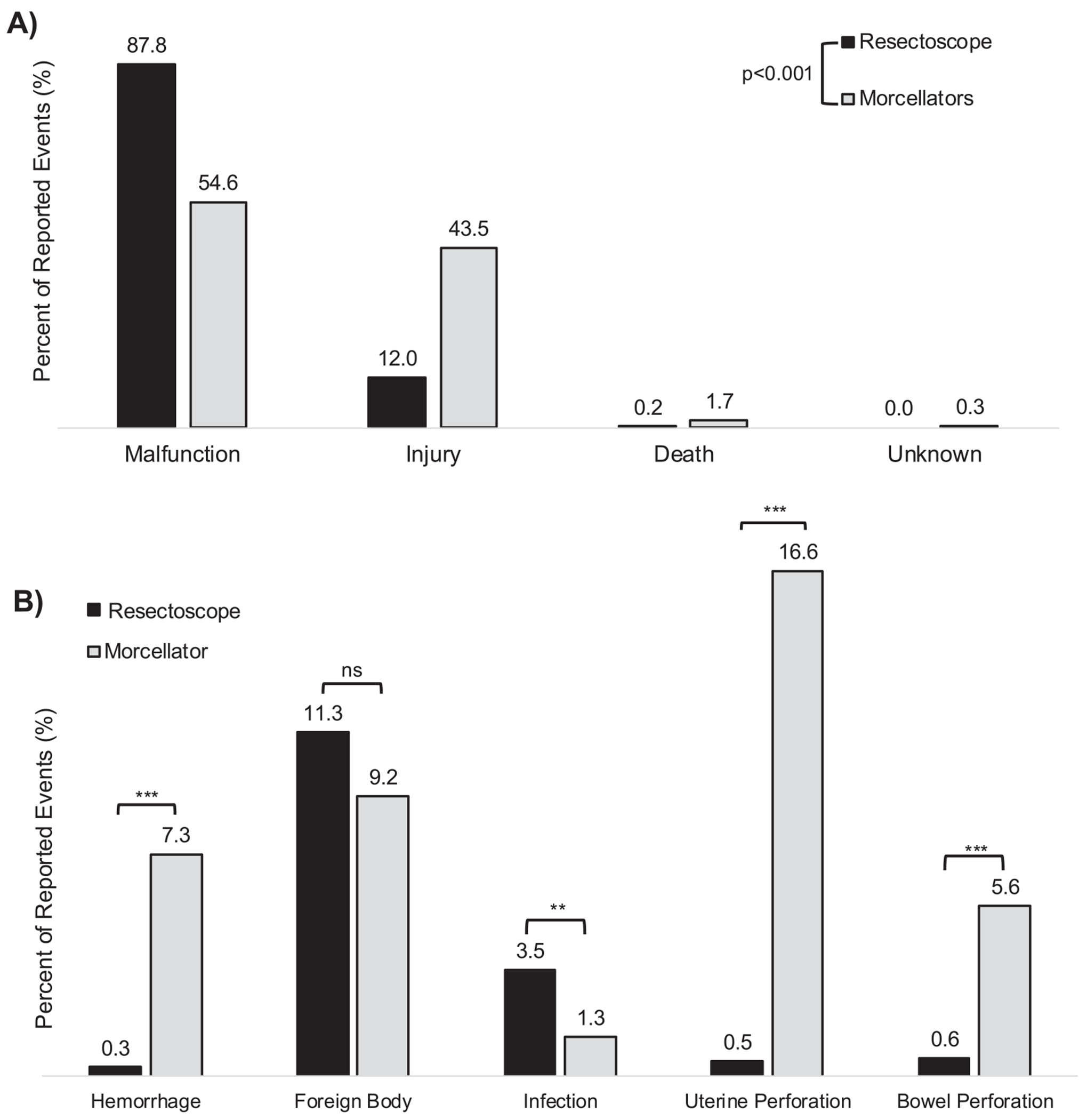
Comparison of AERs based upon device mechanism. (A) Comparison of adverse event type. The MAUDE database will categorize adverse events as malfunction, injury, death, or unknown (if sufficient information is not provided by the reporter). In cases of injury or death, the device is thought to have been a direct factor in patient injury or death. (B) Comparison between the distribution of adverse events that were reported for five specific patient complications with the use of resectoscope vs morcellator devices. *p < .05, **p < .01, ***p < .001.

The distribution of reported adverse event types (e.g., malfunction, injury, etc.) was significantly different between resectoscopes vs hysteroscopic morcellators (TruClear or MyoSure devices) (p < .001) (Fig. 1A). Resectoscopes had a relatively higher frequency of malfunction AERs, while morcellation devices had a relatively higher frequency of injury AERs. Of note, 21 deaths were reported in total from January 2014 to April 2024, 20 of which were reported with hysteroscopic morcellator use.

Each AER was then categorized based on the patient complication reported. For resectoscopes, the majority of AERs involved no patient impact (77.9%), whereas only 45.4% of morcellator AERs reported no patient impact. Impact at the patient level was categorized as: none, foreign body, hemorrhage, burn, infection, air embolism, respiratory distress, hypervolemia, uterine perforation, and bowel perforation (Table 1). Of the 15 burns reported with resectoscope devices, only one event was reported to be secondary to the light cord. The other events were attributed to arcing of the device, malfunction of the working element, or an unknown etiology. For the morcellators, only one of the 6 burn AERs was attributed to the light cord, while the underlying cause of the 5 other events was unknown.

The AER distribution of five common complications between resectoscopes and morcellators were compared. Morcellation devices had relatively higher adverse event reporting for hemorrhage (p < .001), uterine perforation (p < .001), and bowel perforation (p < .001) compared to resectoscopes, whereas resectoscope devices had a higher distribution of reported infection complications (p < .01) compared to morcellators (Fig. 1B). There was not a difference in the reporting of foreign body retention.

The event narrative provided for each AER in the MAUDE database detailed if any further interventions were required. Further medical intervention was disclosed for 2.4% (16/664) of AERs for resectoscopes and 21.1% (255/1208) of AER with hysteroscopic morcellator use. The interventions that were performed were then categorized as operative (including hysterectomy, laparoscopy, laparotomy, or interventional radiology procedure) and nonoperative (including ICU admission, hospitalization, or CPR) (Table 2). While only 1.1% of the reported resectoscope adverse events required operative intervention, 12.4% of the morcellator AERs described operative intervention (p < .001).

Given the significant differences in the reporting of complications and interventions between the use of resectoscopes versus morcellators, it was then questioned if there were differences in safety event reporting between different common brands of hysteroscopic morcellators during this same time period. A subgroup analysis was performed in the same manner, comparing AERs with the TruClear device to AERs with the MyoSure device.

**Fig. 2:**
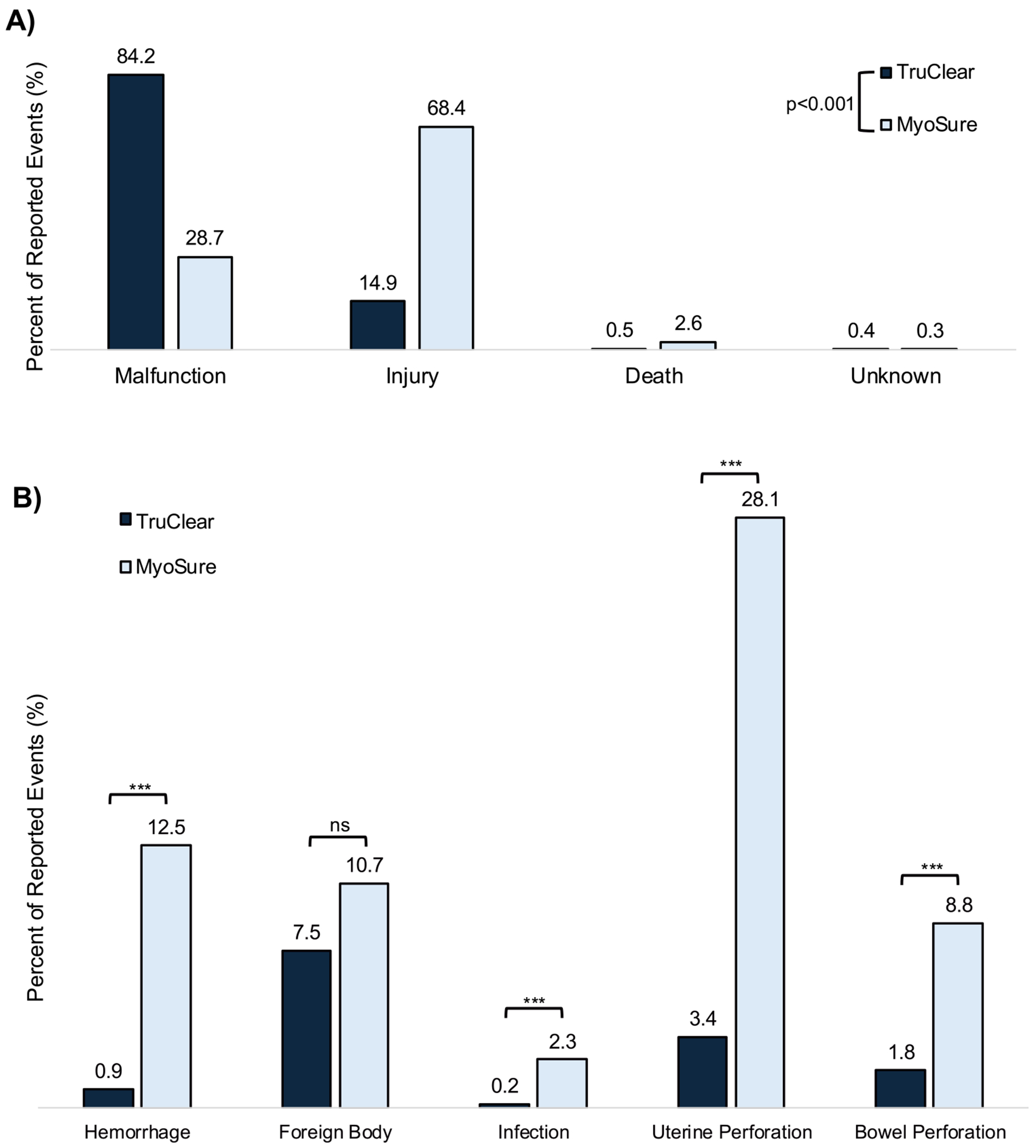
Comparison of AERs between morcellator devices. (A) Comparison of adverse event type (malfunction, injury, death, unknown). Categorization of adverse events described in Fig. 1. (B) Comparison between the distribution of events that were reported for five specific patient complications with the use of both morcellator devices. *p < .05, **p < .01, ***p < .001.

The proportion of adverse event types reported within the MAUDE database was different between TruClear and MyoSure devices: 84.2% (474/563) of TruClear AERs were classified as malfunctions, while 68.4% (441/645) of MyoSure AERs were classified as injuries (Fig. 2A). There was a statistically significant difference in AERs involving direct patient impact for MyoSure compared to TruClear devices (78.8% vs 26.8%, p < .001).

MyoSure device AERs recorded significantly higher frequency of hemorrhage (p < .001), infection (p < .001), uterine perforations (< .001), and bowel perforations (p < .001) compared with TruClear device AERs (Table 3, Fig. 2B). There was no difference in the relative distribution of AERs for foreign body retention with either morcellator device. In examination of subsequent interventions following the index device use, AERs registered with MyoSure devices had a higher distribution of surgical intervention (19.5% vs 4.3%, p < .001) (Table 4).

Though death is a rare complication of hysteroscopy, 21 deaths were recorded within MAUDE as associated with the use of resectoscopes or morcellators from January 2014 to April 2024. Of the 21 deaths, 1 was reported with the use of a resectoscope, 3 reported with TruClear devices, and 17 reported with MyoSure devices (Table S2). The complications associated with death were extrapolated based on independent review of the MAUDE event narratives and included: hemorrhage, cardiac arrest, pulmonary embolism (Table S2). Information on the cause of death was not available for one of the AERs associated with MyoSure device.

## Discussion

Previous studies have suggested that while hysteroscopic resectoscopes and morcellators are both effective devices for the removal of intrauterine pathology, there are not any clearly defined indications or benefits to one device over the other. The current study reports that within the MAUDE database, reporting patterns were different for hysteroscopic devices; there was a significantly lower proportion of reported adverse events of injury and patient harm with the use of resectoscopes compared to morcellators. Furthermore, the current study found differences in the adverse event reporting patterns for two morcellator device brands: MyoSure and TruClear. MyoSure AERs had higher distributions of injuries compared to TruClear, and ultimately had higher distributions of patient complication AERs associated with the need for surgical intervention.

Changes in the manufacturing of devices over the 10-year study period may contribute to differences in the reporting of adverse events with hysteroscopy devices. For example, the TruClear device had been sold to Medtronic from Smith & Nephew in 2017. While there was a transition in the manufacturing of TruClear, the MyoSure device had been acquired by Hologic in 2011, 3 years prior to the study period. Reporting of adverse events may be affected by physical changes to the device in manufacturing or by company-specific protocols.

Differences in device market availability may also contribute to variations in adverse event reporting patterns. The TruClear and MyoSure devices were FDA-cleared in 2005 and 2009, respectively, whereas the first gynecologic resectoscope had been FDA-cleared two decades prior in 1989 [7]. While there is not a way to account for the cumulative experience of the individual surgeon, it should be considered that many of the AERs recorded for the hysteroscopic morcellators may be related to new users in their learning curve or surgeons with lower surgical volume. However, there is evidence that the learning curve with use of hysteroscopic morcellators may be minimal [8], so more recent adoption of this method may not necessarily result in an increased number of AERs.

A previous study utilized the MAUDE database to characterize adverse events reported with TruClear and MyoSure device use from 2005 to 2014 [9]. The current study helps to expand on and update these findings, especially given the increased number of adverse events that were reported for the TruClear device (previous study: 23, current: 563) and MyoSure device (previous study: 87, current: 645). While both the TruClear device and MyoSure device share similarities in their design and operation as mechanical morcellators [10], both studies observed higher distributions of uterine perforation, bowel injury, and surgical intervention with MyoSure AERs within MAUDE compared to TruClear AERs.

While the current study and the previous MAUDE analysis by Haber et al. share similar observations in the comparison of reported adverse events for the TruClear and MyoSure devices, the two studies draw different conclusions regarding the comparison of adverse events occurring with resectoscopes compared to morcellators. Acknowledging the limitations of the MAUDE database, the study by Haber et al. compared the overall adverse event rate for morcellators extrapolated from the MAUDE query to that of adverse events reported with resectoscope device use in a prospective multicenter study [11], to conclude that life-threatening complications occur less frequently with morcellators compared to resectoscopes [9]. The current study used the MAUDE database to compare the reporting patterns (e.g., the proportions of different adverse events types within MAUDE AERs) for resectoscope adverse events vs morcellator adverse events, and ultimately concluded that the reported adverse events associated with morcellator devices more often disclosed life-threatening complications and reported relatively more surgical intervention.

Notably, a small prospective randomized control trial conducted in France noted an increase in complication rates with resectoscopes compared to hysteroscopic morcellators for uterine polyp resection [4]. This randomized prospective study only had 5 operative complications occur within the sample size of 90 patients. As suggested by the study’s authors, the comparison of complication rate between the two devices is difficult to interpret and would require a large sample size, given the overall low complication rate for operative hysteroscopy generally. While the current study is unable to give an estimate for the absolute complication rate for each of the three operative hysteroscopy devices, the large number of adverse events reported through the MAUDE database (resectoscope *N* = 664, TruClear *N* = 563, MyoSure *N* = 645) supports the ability to make the conclusion that reporting patterns of adverse events for resectoscope devices was different from that of morcellation devices. The use of the MAUDE database allows for the evaluation of rare complications that would otherwise require very large sample sizes to have sufficient power.

While the MAUDE database is a large public database that contains some detailed information on reported events, there are significant limitations with its use that are worth noting. One of the strengths of MAUDE is that many different stakeholders (manufacturers, health care professionals, patients, etc.) can file a report. However, despite government legislation that does require reporting of certain complications, the absolute number of adverse events that occur with device use are very likely to be underreported. Adverse events that had little to no impact to patients or seem minimal in the moment but that may still have the potential for harm if the problem is propagated may be less likely to be reported by device users. Furthermore, not all device users may be familiar with the MAUDE reporting system or know how to file a report. Despite the high likelihood that the adverse events included in this study are likely to be just a portion of the absolute number of adverse events that occurred from January 2014 to April 2024, there is no data to suggest that reporting is biased for one device type over another. However, we recognize that weaknesses within the MAUDE database and within its oversight and reporting structure could contribute to bias that affects any conclusions made from direct comparison of devices. Additionally, the number of AERs per device is useful information, however, the interpretation is significantly limited without knowing the total number of each type of procedure performed annually. Without large studies with estimates of the number of hysteroscopy procedures performed annually over the last 5 to 10 years, the estimate of absolute complication rates cannot be made.

With that in mind, while we cannot make conclusions about the differences in utilization among surgeons, there is no available evidence of bias for the reporting adverse events for one device over another, so it is reasonable to draw conclusions, albeit limited, from the comparisons of reporting patterns for each device.

Importantly, the retrospective nature of this study limits the ability to extrapolate potential variables that may underlie the significant differences in reporting patterns for certain adverse event types, including those with patient harm, between operative hysteroscopy devices. For example, details regarding the setting and context of device use are rarely recorded. It is possible that a confounding factor may be the participation of trainees or years of experience that the end user had. Because of the short learning curve associated with hysteroscopic morcellators [8], it is possible that providers with limited hysteroscopic experience utilize this technique over resectoscopic techniques. Furthermore, the level of the provider, or if a provider is at an academic versus private institution, may affect their reporting pattern to MAUDE, which could confound the differences in AERs for the device types. If certain types of users are more likely to use MyoSure devices, it may be the characteristics of the user that are really associated with adverse events, rather than the instrument used. For example, if more experienced users or higher-volume surgeons are more likely to use TruClear and more novice users are more likely to use MyoSure, it is plausible that it is user experience that is associated with a specific adverse event identified, rather than MyoSure itself. Additionally, distinct uterine pathologies may be associated with particular complications. While some of the event narratives specifically mentioned the indication for the use of the operative hysteroscopy devices, this detail was not universally available. The resectoscope has the ability to resect fibroids with an intramural component, which is a more complex surgery than polypectomy or treatment of a FIGO Type 0 or 1 fibroid. As such, the lower number of event reports with the resectoscope (in spite of use in possibly more complex surgeries) may reflect use by surgeons with greater skill. Different MyoSure devices (e.g., Myosure XL vs Myosure Reach vs Myosure Light) are designed to be utilized for different uterine pathologies. Without having details on the specific MyoSure device used for all adverse events, this study was unable to categorize all AERs by the specific device used; accordingly, the decision was made to group the mechanical morcellator devices by brand, as the underlying mechanism is the same across the models. However, it is possible that the adverse events reported could be confounded by the specific device used, given the targeted intrauterine pathology. A prospective study design would allow for the investigation of additional factors that contribute an increased risk of significant patient complications.

## Conclusions

This secondary analysis of a publicly available FDA database demonstrates significant differences in the reporting patterns for adverse events associated with the use of operative hysteroscopy instruments. AERs for morcellation devices had a higher distribution of significant and substantial patient impact and operative interventions compared to AERs for hysteroscopic resectoscope devices. On further subgroup analysis of morcellators, reports with MyoSure devices had a significantly higher distribution of serious patient complications (uterine/bowel perforation, infection, hemorrhage) compared to TruClear device AERs. Findings from this study would best be re-examined in a prospective study.

While the use of a secondary database with inconsistent reporting may contribute bias, which limits the scope of the conclusions made, this study calls attention to the significant risks that are associated with operative hysteroscopy overall. While our statistical analysis cannot provide information pertaining to the absolute rate of adverse events per unit case or per unit instrument sold, the differing proportions of adverse events with each device may suggest a different general risk profile, and at the very least, a different reporting pattern when comparing each device type. This could ultimately impact providers’ consent process for operative hysteroscopy to a more device-specific consenting approach. Ultimately, it is imperative that device users recognize the importance of complete and detailed reporting for all adverse events for all devices, as reporting patterns are different between our studied devices. In doing so, the associated risks of specific devices—albeit with similar functions and utilized for the same pathologies—may be better characterized.

## Supplementary Material

1

## Figures and Tables

**Table 1:** Comparison of patient complications by operative hysteroscope device type

	Resectoscope (*N* = 664) *N*, (%)	Morcellator (*N* = 1208) *N*, (%)
**None**	517 (77.9)	549 (45.4)
**Foreign body**	75 (11.3)	111 (9.2)
**Hemorrhage**	2 (0.3)	88 (7.3)
**Burns**	15 (2.3)	6 (0.5)
**Uterine perforation**	3 (0.5)	200 (16.6)
**Bowel perforation**	4 (0.6)	67 (5.6)
**Infection**	23 (3.5)	16 (1.3)
**Tissue injury**	4 (0.6)	25 (2.1)
**Hyper/hypovolemia**	1 (0.2)	17 (1.4)
**Air embolism**	3 (0.5)	2 (0.2)
**Pulmonary embolism**	0	2 (0.2)
**Electrolyte imbalance**	0	1 (0.1)
**Respiratory distress**	0	14 (1.2)
**Cardiac issue (arrythmia, cardiac arrest)**	0	8 (0.7)
**Seizures/convulsions**	0	2 (0.2)
**Cancer dissemination**	0	4 (0.3)
**Insufficient information**	17 (2.6)	96 (8.0)

Complications recorded within AERs attributed to the use of either resectoscope or morcellator devices.

**Table 2:** Comparison of postcomplication interventions by operative hysteroscope device type

	Resectoscope (*N* = 664) *N*, (%)	Morcellator (*N* = 1208) *N*, (%)
**None**	648 (97.6)	953 (78.9)
**Operative intervention**	7 (1.1)	150 (12.4)
**Nonoperative intervention**	2 (0.3)	105 (8.7)
**Unknown**	7 (1.1)	0

Interventions that occurred within AERs associated with resectoscope or morcellator device use. Medical interventions included hospitalization, ICU admission, and CPR. Operative interventions included diagnostic laparoscopy, exploratory laparotomy, hysterectomy, and interventional radiology procedures. The narratives for seven of the resectoscope AERs mentioned need for intervention, but did not specify what interventions were performed.

**Table 3:** Comparison of patient complications for types of hysteroscopic morcellator devices

	TruClear (*N* = 563) *N*, (%)	MyoSure (*N* = 645) *N*, (%)
**None**	412 (73.2)	137 (21.2)
**Foreign body**	42 (7.5)	69 (10.7)
**Hemorrhage**	5 (0.9)	83,(12.5)
**Burns**	3, (0.5)	3 (0.5)
**Uterine perforation**	19 (3.4)	181 (28.1)
**Bowel perforation**	10 (1.8)	57 (8.8)
**Infection**	1 (0.2)	15 (2.3)
**Tissue injury**	3 (0.5)	22 (3.4)
**Hyper/hypovolemia**	2 (0.4)	15 (2.3)
**Air embolism**	0	2 (0.3)
**Pulmonary embolism**	0	2 (0.3)
**Electrolyte imbalance**	0	1 (0.2)
**Respiratory distress**	1 (0.2)	13 (2.0)
**Cardiac issue (arrythmia, cardiac arrest)**	2 (0.5)	6 (0.9)
**Seizures/convulsions**	0	2, (0.3)
**Cancer dissemination**	1 (0.2)	2 (0.3)
**Insufficient information**	62 (11.0)	34 (5.3)

Complications recorded within AERs attributed to the use of either the Tru-Clear device or MyoSure device.

**Table 4:** Comparison of postcomplication interventions by morcellator type

	TruClear (*N* = 563) *N*, (%)	MyoSure (*N* = 645) *N*, (%)
**None**	529 (94.0)	424 (65.7)
**Operative intervention**	24 (4.3)	126 (19.5)
**Nonoperative intervention**	10 (1.8)	95 (14.7)

Interventions that occurred within AERs associated with TruClear or MyoSure device use. Medical interventions included hospitalization, ICU admission, and CPR. Operative interventions included diagnostic laparoscopy, exploratory laparotomy, hysterectomy, and interventional radiology procedures.

## Data Availability

Data is available from the authors upon reasonable request.

## References

[1] Hamerlynck TWO, van Vliet HAAM, Beerens AS, Weyers S, Schoot BC. Hysteroscopic morcellation versus loop resection for removal of placental remnants: a randomized trial. J Minim Invasive Gynecol. 2016;23:1172–1180. 27590568 10.1016/j.jmig.2016.08.828

[2] Yin X, Cheng J, Ansari SH, Hysteroscopic tissue removal systems for the treatment of intrauterine pathology: a systematic review and meta-analysis. Facts Views Vis Obgyn. 2018;10:207–213. 31367293 PMC6658200

[3] Ren F, Huang G, Wang X, Li X, Cai J. Comparison of hysteroscopic morcellation versus resectoscopy in treatment of patients with endometrial lesions: a meta-analysis. Med Sci Monit. 2022;28:e936771. 35844074 10.12659/MSM.936771 PMC9306303

[4] Stoll F, Lecointre L, Meyer N, Randomized study comparing a reusable morcellator with a resectoscope in the hysteroscopic treatment of uterine polyps: the RESMO study. J Minim Invasive Gynecol. 2021;28:801–810. 32681995 10.1016/j.jmig.2020.07.007

[5] Noventa M, Ancona E, Quaranta M, Intrauterine morcellator devices: the icon of hysteroscopic future or merely a marketing image? A systematic review regarding safety, efficacy, advantages, and contraindications. Reprod Sci. 2015;22:1289–1296. 25878200 10.1177/1933719115578929

[6] Hysteroscopy Procedures Market Size & Share Report, 2030. Available at: https://www.grandviewresearch.com/industry-analysis/hysteroscopy-procedures-market-report. Accessed October 16, 2024, n.d.

[7] Vitale SG, Giannini A, Carugno J, Hysteroscopy: where did we start, and where are we now? The compelling story of what many considered the “Cinderella” of gynecological endoscopy. Arch Gynecol Obstet. 2024;310:1877–1888. 39150502 10.1007/s00404-024-07677-x PMC11393125

[8] van Dongen H, Emanuel Mark MH, Wolterbeek R, Trimbos JB, Jansen FW. Hysteroscopic morcellator for removal of intrauterine polyps and myomas: a randomized controlled pilot study among residents in training. J Minim Invasive Gynecol. 2008;15:466–471. 18588849 10.1016/j.jmig.2008.02.002

[9] Haber K, Hawkins E, Levie M, Chudnoff S. Hysteroscopic morcellation: review of the manufacturer and user facility device experience (MAUDE) database. J Minim Invasive Gynecol. 2015;22:110–114. 25128851 10.1016/j.jmig.2014.08.008

[10] Franchini M, Ceci O, Casadio P, Mechanical hysteroscopic tissue removal or hysteroscopic morcellator: understanding the past to predict the future. A narrative review. Facts Views Vis Obgyn. 2021;13:193–201. 34555873 10.52054/FVVO.13.3.026 PMC8823270

[11] Jansen FW, Vredevoogd CB, van Ulzen K, Hermans J, Trimbos JB, Trimbos-Kemper TC. Complications of hysteroscopy: a prospective, multicenter study. Obstet Gynecol. 2000;96:266–270. 10908775 10.1016/s0029-7844(00)00865-6

