# Supplementary material for: Food and Drug Administration Database Secondary Analysis: Difference in Operative Hysteroscopy Device Adverse Event Reporting
Source: J Minim Invasive Gynecol. Author manuscript; Available in PMC 2025 Dec 11. (PMC12696751; doi:10.1016/j.jmig.2025.04.009)
Supplement: 1 [file NIHMS2123169-supplement-1.pdf]

## Supplemental Table 1

Reporting of adverse events by device type

| | Resectoscope ($N = 664$) | TruClear ($N = 563$) | MyoSure ($N = 645$) |
|---|---|---|---|
| **2014** | 24 | 11 | 51 |
| **2015** | 30 | 75 | 61 |
| **2016** | 19 | 41 | 71 |
| **2017** | 48 | 189 | 66 |
| **2018** | 41 | 70 | 50 |
| **2019** | 24 | 32 | 62 |
| **2020** | 32 | 39 | 50 |
| **2021** | 30 | 32 | 81 |
| **2022** | 50 | 36 | 71 |
| **2023** | 322 | 36 | 71 |
| **2024** | 44 | 2 | 11 |

The number of adverse events for each device type that were reported from 2014 to April 2024. The events are listed and organized by the year the event was reported. All three devices had at least two adverse events reported for every year included in the MAUDE search query. Resectoscope $N = 664$, TruClear $N = 563$ events, MyoSure $N = 645$.

## Supplemental Table 2

Deaths reported from 2014 to 2024

| | Resectoscope ($N = 1$) | TruClear ($N = 3$) | MyoSure ($N = 17$) |
|---|---|---|---|
| **Cardiac arrest** | 0 | 2 | 2 |
| **Air embolism** | 1 | 0 | 0 |
| **Cancer dissemination** | 0 | 1 | 3 |
| **Hemorrhage** | 0 | 0 | 1 |
| **Uterine perforation** | 0 | 0 | 4 |
| **Bowel perforation** | 0 | 0 | 3 |
| **Pulmonary edema** | 0 | 0 | 2 |
| **Pulmonary embolism** | 0 | 0 | 1 |
| **No information** | 0 | 0 | 1 |

21 deaths were reported to be associated with adverse events for the three operative hysteroscopy devices studied. Deaths are organized based upon which device the event was reported with and what patient complication was reported for that particular event.

Downloaded for Anonymous User (n/a) at JOHNS HOPKINS UNIVERSITY from ClinicalKey.com by Elsevier on November 14, 2025. For personal use only. No other uses without permission. Copyright ©2025. Elsevier Inc. All rights reserved.
